# Effect of Sequence of Fruit Intake in a Meal on Satiety

**DOI:** 10.3390/ijerph16224464

**Published:** 2019-11-13

**Authors:** Bibi Nabihah Abdul Hakim, Hanis Mastura Yahya, Suzana Shahar, Zahara Abdul Manaf, Hanafi Damanhuri

**Affiliations:** 1 Dietetics Programme, Centre for Healthy Aging & Wellness, Faculty of Health Sciences, Universiti Kebangsaan Malaysia, Kuala Lumpur 50300, Malaysia; bibinabihah@yahoo.com.my (B.N.A.H.); suzana.shahar@ukm.edu.my (S.S.); zaharamanaf@ukm.edu.my (Z.A.M.); 2 Nutritional Science Programme, Centre for Healthy Aging & Wellness, Faculty of Health Sciences, Universiti Kebangsaan Malaysia, Kuala Lumpur 50300, Malaysia; 3 Department of Biochemistry, Faculty of Medicine, UKMMC, Universiti Kebangsaan Malaysia, Jalan Yaacob Latif, Cheras, Kuala Lumpur 56000, Malaysia; hanafi.damanhuri@ppukm.ukm.edu.my

**Keywords:** fruit, sequence, satiety, energy intake, blood glucose

## Abstract

Little is known about the effects of manipulating sequence of fruit consumption during a meal in suppressing an individual’s appetite. Therefore, we investigate the effects of the sequence of fruit intake on satiety and blood glucose in a group of 17 healthy, young male adults. This intervention study repeatedly measured the effects of fruit intake (120 g red apple) before and after a meal and control (no fruit). Ad libitum test meal was weighed before and after a meal. Subjective appetite rating and appetite-related hormones were assessed at regular time intervals. The satiety score was significantly higher for fruit intake before a meal followed by after a meal and control (*p* < 0.05). Eating fruit before a meal reduced 18.5% (166 kcal) subsequent energy intake compared to control (*p* < 0.05). Fruit intake before a meal had a significantly higher incremental area under the curve (iAUC) of Glucagon-like peptide 1 (GLP-1), compared to after a meal (*p* < 0.05). There were no differences in plasma changes of ghrelin, Cholecystokinin 8 (CCK8), or blood glucose in all sessions. Consuming fruit before a meal potentially enhanced satiety. Further research is required to confirm both short- and long-term effects of the sequence of fruit intake on appetite regulation in a wider population.

## 1. Introduction

Natural appetite suppressant foods have been found to exploit regulatory mechanisms that control feeding habits [1,2]. This new approach has gained widespread interest and has longer-lasting effects [3,4]. It promotes reduction in food intake, whilst aiding with compliance by reducing the sensation of hunger [5,6]. As part of a daily diet, fruits have been found to have potential benefits in suppressing appetite, with low energy density, less fat, and high water content with a considerable amount of dietary fiber [7]. Incorporating whole fruits in daily diet helps an individual feel fuller in lower calories and eat less in the subsequent meal. 

Consuming fruit before a meal, regardless of different forms, led to a greater reduction of hunger and food intake than without preload [8]. Trico et al. [9] found that manipulating the sequence of food intake enhanced satiety and optimized glycemic control. Preload of lipids and proteins has been shown to have a positive effect on glucose tolerance and help with delaying gastric emptying. A previous study also reported a significant impact of manipulating macronutrient order during a meal on postprandial glucose and insulin excursions as well as secretion of gut hormones [10]. The carbohydrate-last meal pattern stimulated lower postprandial glucose compared to a carbohydrate-first meal and sandwich (all meal components were eaten together) [11]. However, little is known on the effect of manipulating the sequence of fruit consumption on satiety and its hormone regulation and blood glucose control. 

Most health professionals address the negative impact of sugar content in fruit on glycemic control and, therefore, diabetic patients or those with impaired glucose tolerance have been advised to restrict their fruit intake to a maximum of two pieces a day [12]. In a clinical setting, the suggestion for the best time to consume fruit was based on expert opinion only [13]. In fact, culturally, fruits are served after a meal and regarded as desserts. 

Therefore, we aimed to determine whether consumption of fruit in a different order, either before a meal or after a meal, will affect satiety, food intake, and blood glucose concentration. It was hypothesized that consumption of fruit before a meal would have a higher suppressive effect on appetite, reduce food intake, and improve blood glucose. The findings highlight the implications toward the best sequence of consuming fruit in controlling appetite and blood glucose level. 

## 2. Materials and Methods 

### 2.1. Study Population

One hundred and twelve male subjects from a phase 1 study, which was published elsewhere [14], were screened based on inclusion and exclusion criteria. Forty young adults, between 20 and 39 years old, who fulfilled the following inclusion criteria: Male, healthy with no chronic diseases, body mass index (BMI) 18.5–24.9 kg/m^2^, not a regular consumer of vitamin or mineral supplements in the past 6 months, were selected. Subjects were excluded if they had planned to gain or lose weight in the past 6 months, had gastrointestinal discomfort, wore braces, drank 4 or more servings of caffeinated drink daily (equivalent to 300–400 mg/day), smoker, drank alcohol, were athletes, body builders, or had food allergies. The subjects were then assessed for metabolic profile (Hemoglobin A1c (HbA1c), fasting blood sugar (FBS), lipid profile, hemoglobin, renal function test, and liver function test). Potential subjects completed the Hospital Anxiety and Depression Scale (HADS) (measures symptoms of depression and anxiety) [15] and Three-Factor Eating Questionnaire-R21 (TFEQ-R21), a 21-item version questionnaire [16] for eating attitude assessment, which evaluates cognitive restraint, emotional eating, and uncontrolled eating. These measurements were used to ensure there was no symptom of depression and anxiety or disordered eating, which might influence the study outcome. The final number of subjects who fulfilled the criteria from phase I and other recruitment is 20. This is to control the effect of intervention since this metabolic profile has been shown to affect regulation of appetite-related hormone [17]. Before intervention, a 3-day diet record was obtained to evaluate their habitual energy intake. Written informed consent was obtained from all subjects, and subjects were financially compensated for their participation. The study was in accordance with the Declaration of Helsinki, and the protocol was approved by the Universiti Kebangsaan Malaysia Medical Research and Ethics Committee (NN-2016-032). 

### 2.2. Foods and Beverages

A serving of non-peeled Red Delicious apple (Washington), weighing 120 g containing approximately 60 kcal energy with 2.88 g of fiber, was cut in wedges. Providing one serving of fruit at a meal was based on recommendation by Malaysian Dietary Guideline [18]. The test meal consisted of 650 g of fried rice (1200 kcal) served with 600 mL of plain water (at room temperature). Portion sizes were based on lunch intake from dietary record in previous lunch intake data and provided higher calories than most subjects were likely to consume [8]. The test meal contained 49.6% energy from carbohydrate, 10.5% energy from protein, and 39.9% energy from fat. All foods and beverages were weighed before and after a meal to the nearest 0.1 g to determine the amount of food and beverage consumed [8]. Energy intake was calculated using Nutritionist Pro™ Diet (Axxya Systems-Nutritionist Pro, Stafford, TX, USA).

### 2.3. Experimental Design

**Figure 1:**
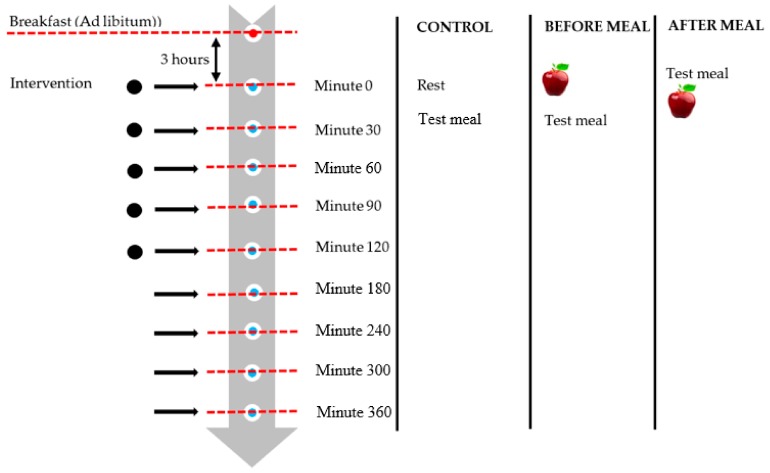
Study design. 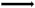 Measurement of subjective appetite rating; 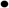 Blood samples.

This intervention study consists of three consecutive sessions separated by a seven-day wash out period (Figure 1). The sessions included fruit intake before a meal, after a meal, and control (no fruit). Prior to each session, the subjects were asked to fast overnight for 10–12 h. Subjects were also asked to ensure that their dietary intake and physical activity was as consistent as possible across the sessions. 

On test days, each subject was seated alone in a different room with the same conditions. The compliance and health status of the subjects were evaluated prior to each session to ensure they were feeling well, or they would be rescheduled. Each subject was served a standard breakfast of fried rice noodles and tea ad libitum to ensure a consistent level of hunger before starting the intervention. 

Lunch was scheduled 3 h after breakfast. Within this three-hour interval, subjects had to avoid taking in any foods or drinks. Only plain water was permitted between meals until one hour prior to each test session. During the control session, at minute 0, no fruit was served. The subjects were provided with books and magazines and were asked to sit quietly. The test meal was served at minute 30. The subjects were given up to 20 min to complete the test meal ad libitum until they reached comfortable satiation [9]. The time taken to consume the test meal and the liking score of the test meal were recorded for each subject. For the following session, subjects were required to finish the given fruit at minute 0, followed by the test meal at minute 30 [19]. For the last session, the subjects were given their test meals 30 min before the fruit was served. Subjects were asked to record their post-meal intake in the provided food diary to assess their subsequent meal intake and total daily energy intake. The subjects were instructed to consume only provided fruit during the day of intervention. Three-day diet recall was recorded during the wash out period to ensure adherence to the study protocol. 

### 2.4. Subjective Appetite Rating

Appetite sensations were assessed using a validated series of 100 mm visual analogue scale (VAS) [20] before and after breakfast, at minute 0 and in 30-min intervals up to 120 min. The subjective assessment consisted of four ratings, and included “hunger”, “fullness”, “satiation”, and “desire to eat” anchored by “not at all” on the left side and “extremely” on the right side. These four appetite ratings were then summarized into one score, named the composite satiety score (CSS). The composite satiety score is a global score of appetite sensation. The formula below was adapted from previous research [21,22,23]. A higher score indicates a greater satiety level. The CSS was calculated individually by using the following formula:

CSS = (satiety + fullness + (100 − prospective food consumption) + (100 − hunger))/4.

### 2.5. Blood Analysis

Venous blood samples were collected at minute 0 and in 30-min intervals up to 120 min. Each blood sample was collected into two different EDTA tubes, labelled tube A and tube B. A total of 30 uL of DPP-IV inhibitor was added to tube A containing 3 mL of blood sample to prevent degradation of GLP-1 hormone. Tube A was used for analysis of plasma GLP-1 whilst tube B contained 5 mL of blood for analysis of plasma ghrelin and CCK8. All the EDTA tubes were placed on ice before centrifugation. The blood sample in tube A was separated by centrifugation at 2000 *g* for 15 min at 4 °C and tube B was centrifuged at 1000 *g* for 15 min at 4 °C. The aliquots were stored at −80 °C until analysis. Human enzyme-linked immunosorbent assay (ELISA) kits were used to measure plasma ghrelin (Elabscience, Wuhan, China), CCK8 (Elabscience, Wuhan, China), and GLP-1 (Millipore, Missouri, MO, USA). Blood glucose was measured using a glucometer. 

### 2.6. Power Calculation

According to power analysis, a sample size of 16 was calculated using formula calculation by Noordzij et al. [24]. It would be sufficient to detect a 50 kcal difference in energy intake during the test meal at a significant level of 0.05 with 80% of power. Considering 20% additional subjects to allow adjustment of withdrawal, a total of 20 subjects were recruited in this study.

### 2.7. Data Analysis

All data were analyzed using Statistical Package for Social Sciences (SPSS) software version 21.0 (IBM Corporation, Armonk, New York, NY, USA). Data were tested for normality prior to analysis. The mean differences of composite satiety score (CSS), energy intake, blood glucose, and plasma hormonal changes were analyzed using one-way repeated measure ANOVA. Post hoc analysis using Bonferroni was conducted when the treatment effect was significant. The incremental area under the curve (iAUC) or over the curve (iAOC) was calculated using the trapezoidal method. The time-average iAUC or iAOC was divided by time to provide a mean value for 120 min intervention. Multiple linear regression was applied to assess the confounding factor of the intervention. The correlation between subjective appetite rating and blood glucose concentration with appetite-related hormone were assessed by using Pearson’s correlation coefficient. The results are presented as mean ± standard error. The significant value was set at *p* < 0.05.

## 3. Results

A total of 17 healthy young male adults, the majority of whom were college students, with normal body mass index (21.2 ± 1.5 kg/m^2^) were involved in this study. Two subjects dropped out during the second session due to time constraint and one subject was excluded due to gaining weight and was classified as overweight during the third session. 

**Figure 2:**
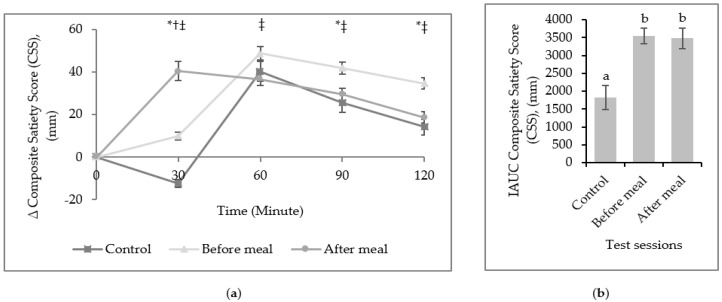
(**a**) Mean (± SEM) change (∆) in Composite Satiety Score (CSS). * Significant difference between control and before a meal, *p* < 0.05, † significant difference between control and after a meal, *p* < 0.05, ‡ significant difference between before and after a meal, *p* < 0.05 with one-way repeated measure ANOVA, Bonferroni adjusted-pairwise comparison. (**b**) Incremental area under the curve (iAUC) of CSS across the lunch meal. Different letter denotes significantly different (*p* < 0.05) with one-way repeated measure ANOVA, Bonferroni adjusted-pairwise comparison.

The significant difference of changes in postprandial CSS appeared to be at minute 30. A greater increment of CSS was observed for after a meal compared to before a meal, and reduction of CSS was observed during control, *p* < 0.05. At minute 60, before a meal had a higher increment of CSS compared to after a meal. There was a greater increment of CSS during before a meal compared to after a meal and control at 90- and 120-min time points, *p* < 0.05. Overall, consumption of fruit before a meal (3544 ± 907 mm) and after a meal (3478 ± 1210 mm) led to a significantly higher score of iAUC CSS (*p* < 0.0001) compared to control (1817 ± 1386 mm) (Figure 2).

The energy intake during the test meal did not differ in all three sessions, nor for total daily energy intake. However, our study reported a significant 18.5% decreased energy intake (166 kcal) during the subsequent meal for before a meal as compared to control, *p* < 0.05 (Table 1). Analysis using a multiple linear regression test confirmed that there was no significant effect from confounding factors such as the hedonic ratings of foods, time taken in consuming the meal, and fluid intake on energy intake in all three sessions.

**Figure 3:**
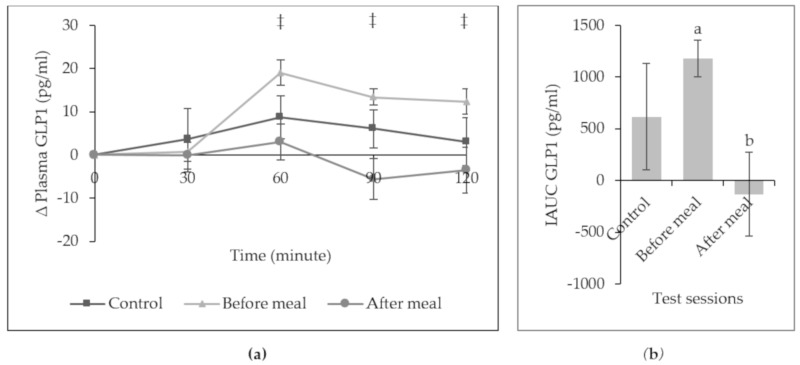
(**a**) Mean (± SEM) change (∆) in plasma GLP-1. * Significant difference between control and before a meal, *p* < 0.05, † significant difference between control and after a meal, *p* < 0.05, ‡ significant difference between before and after a meal, *p* < 0.05 with one-way repeated measure ANOVA, Bonferroni adjusted-pairwise comparison. (**b**) Incremental area under the curve (iAUC) of plasma GLP-1 across the lunch meal. Different letter denotes significantly different (*p* < 0.05) with one-way repeated measure ANOVA, Bonferroni adjusted-pairwise comparison.

Figure 3 illustrates the postprandial response of plasma GLP-1 over 120 min. There was a prompt rise of plasma GLP-1 at minute 60 for all sessions. Fruit intake before a meal had a greater increment of plasma GLP-1 as compared to after a meal at minute 60, 90, and 120 min. Overall, the iAUC of plasma GLP-1 for fruit intakes before a meal (1178.751 ± 725.20 pg/ml) was significantly higher than those fruit intake after a meal (−131.934 ± 1660.71 pg/ml), (*p* = 0.003).

**Figure 4:**
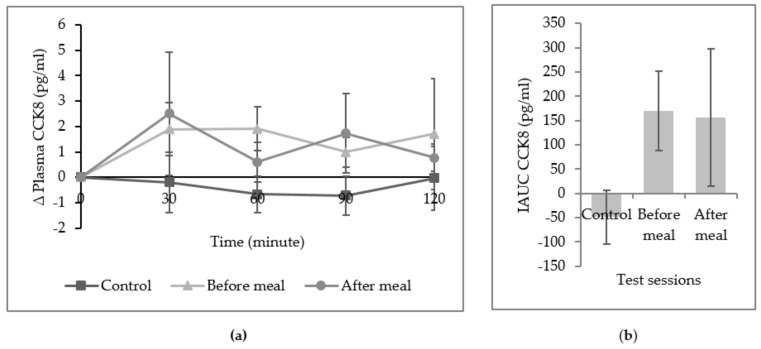
(**a**) Mean (± SEM) change (∆) in plasma CCK8. (**b**) Incremental area under the curve (iAUC) of plasma CCK8 across the lunch meal. No significant difference was found in all three sessions with one-way repeated measure ANOVA, Bonferroni adjusted-pairwise comparison.

**Figure 5:**
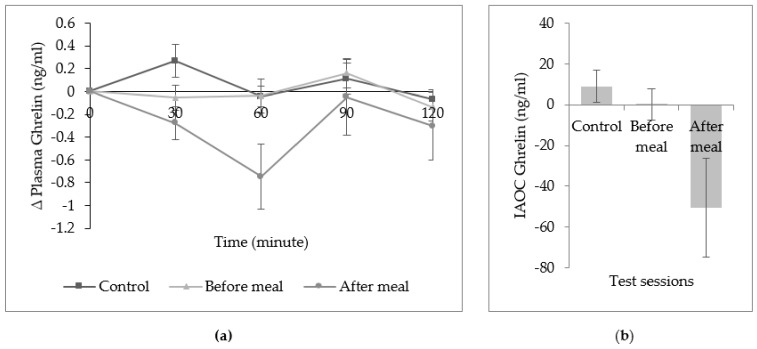
(**a**) Mean (± SEM) change (∆) in plasma ghrelin. (**b**) Incremental area over the curve (iAOC) of plasma ghrelin across the lunch meal. No significant difference was found in all three sessions with one-way repeated measure ANOVA, Bonferroni adjusted-pairwise comparison.

The study confirmed that there was no mean difference of plasma CCK8 in all three sessions as well as for iAUC of plasma CCK8 (Figure 4). Figure 5 also demonstrated a non-significant change of plasma ghrelin from baseline at all time point. Overall, the incremental area over the curve of plasma ghrelin did not differ in all sessions.

Table 2 highlights the relationship between subjective appetite rating and plasma changes of appetite-related hormone. Subjective appetite rating had no significant relationship with hormonal changes reported by subjects in all three sessions.

**Figure 6:**
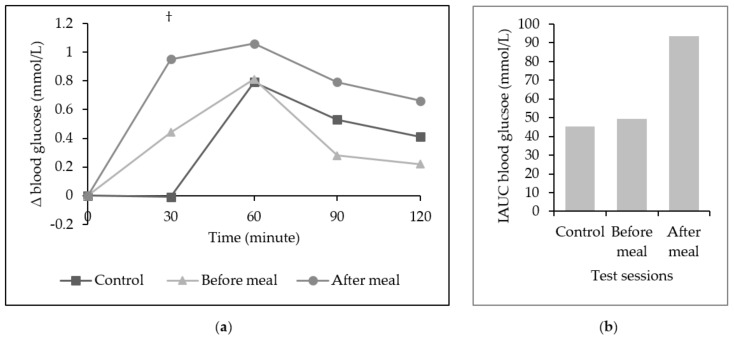
(**a**) Mean (± SEM) change (∆) in blood glucose level. † Significant difference between control and after a meal, *p* < 0.05 with one-way repeated measure ANOVA, Bonferroni adjusted-pairwise comparison. (**b**) Incremental area under the curve (iAUC) of blood glucose across the lunch meal. No significant difference was found in all three sessions with one-way repeated measure ANOVA, Bonferroni adjusted-pairwise comparison.

The mean changes in blood glucose level were not significantly different in all three sessions at all time points except at minute 30 (Figure 6). A higher increment of blood glucose level was reported during the after a meal session as compared to control. Our study also found no significant difference in iAUC of blood glucose for control, before a meal, or after a meal, *p* > 0.05.

## 4. Discussion

This is the first study of its kind, which objectively examined the effect of manipulating the sequence of fruit intake on appetite sensation, energy intake, hormone regulation, and blood glucose control. Consumption of fruit before a meal was associated with a greater appetite score, reduced subsequent energy intake, and increased appetite-related hormone (GLP-1). 

An earlier study also reported higher satiety levels after preload consumption as compared to control (no fruit) despite different forms of fruits [8]. A previous study also found a stronger satiety effect of prune preload as compared to a preload of an isocaloric bread product [25]. The suppressive effect on subjective rating was partly supported by the increment of plasma GLP-1. A higher increment of plasma GLP-1 at minute 90 and 120 also showed a relative effect on short-term satiety of fruit consumption before a meal as compared to after a meal. The pre-exposure of fiber content in fruit may explain its benefits in suppressing appetite and prolonging satiety [8] as compared to consumption of fruit after a meal. Fruit rich in fiber enhances satiety by increasing the effort for mastication and initiates cephalic phase responses [25,26], thus stimulating production of gut hormones [27]. The production of the GLP-1 hormone slows the gastric emptying and makes individuals feel full for longer [28]. Shukla et al. [10] also reported a significant increment of plasma GLP-1 for those that consumed protein and vegetables before a carbohydrate meal as compared to after a carbohydrate meal. 

However, manipulating the sequence of fruit intake did not affect energy intake during the lunch test meal. A previous study reported an opposite effect, in which consumption of fruit before a meal was associated with reduction of energy intake during the test meal as compared to no preload [8]. Houchins et al. [29] also found a significant reduction of energy intake during the test meal after consumption of fruit compared to no preload. The discrepancy could be due to the small portion size of fruit served in the present study whilst others provided two or more servings of fruit in their studies [8,25,29]. Furthermore, this variability of outcomes may also be due to a longer inter-meal interval (30 min) between consumption of fruit and the test meal in our study as compared to the studies by Flood Obbagy and Rolls [8], which was 15 min, and Houchins et al. [29], which was just before a meal; albeit, a time lapse of 30 min has been recommended to investigate gastrointestinal and satiety effects [30]. Previous published work also recommended an inter-meal interval between 30 to 120 min to maximize the energy compensation of semisolid/solid preload [31].

Furthermore, our present study observed a postprandial satiety effect as there was a significant 18.5% reduction of subsequent energy intake following fruit intake before a meal as compared to control. In contrast, an isoenergetic snack of dried plums had no effect on ad libitum energy intake, compared to low-fat cookies at a meal two hours later [32]. The previous study also failed to obtain a significant result where subsequent energy intake did not differ in all three meals (added avocado, inclusive avocado (avocado was added and matched with macronutrient content in control test meal), and no avocado) due to a 5-h time interval between the lunch test meal and the ad libitum dinner meal [33]. The previous study reported that the commencement of preload consumption may affect subsequent appetite and food intake for only up to two hours after preload [34]. 

The temporal sequence of fruit intake during a meal, however, did not affect the plasma changes of ghrelin and CCK8. The non-significant finding in our study might be due to the lower fiber content served as compared to the previous study. Kaliora et al. [35] demonstrated a significant reduction of plasma ghrelin after consumption of raisin containing 5 g of fiber as compared to glucose. In addition, previous study also found a significant increment of plasma CCK after consumption of fiber-rich food containing 12 g fiber [36].

No association was found in our study between subjective appetite rating and appetite-related hormone. Our study only found a significant inverse relationship between CSS and ghrelin during control. It was found that fiber content from fruit may influence the changes in the appetite-related hormone through the chewing process [8]. The appetite-related hormone was based on individual perception whilst changes in hormone was also affected by other factors (early post-ingestive cues and chewing process). Since no fruit was provided in control session, there would be no confounding effect (related to early post-ingestive cues and chewing process) that would influence the changes in the appetite-related hormone (ghrelin). Lobley et al. [37] explained that the self-reported appetite ratings cannot be used to predict the changes of appetite-related hormone as they are only based on individual interpretation. The different response of hormonal changes may also have been affected by other factors. These include cognitive and sensory factors (such as expectations of satiety value, or visual and oral cues about the amount of food), as well as early post-ingestive cues such as gastric distension [38].

An individual’s appetite may also be affected by such factors as gender differences, physical activity, sleep duration, sensory characteristic of food intake, and duration of food and fluid intake during the test meal. A previous study found a higher appetite suppressive effect among women as compared to men. This might be due to the physiological regulation of appetite through sex hormones [39]. Sleep duration [40] and body weight [41] have been found to be important regulator of one’s appetite. Different levels of physical activity may also influence appetite through modulation of hunger and adjustment of postprandial satiety through interaction between food compositions [42]. These three factors have been controlled in our present study and therefore the risk of bias was reduced. In addition, our study found no significant difference of sensory characteristic of fruit intake, duration of food intake, and fluid intake during the test meal amongst all subjects in all intervention periods.

This study also demonstrated that manipulating the sequence of fruit consumption had no effect on postprandial blood glucose. A significantly higher increment of blood glucose level was observed only at minute 30, which might be due to consumption of test meal during the session of after meal as compared to control where the subjects were seated without consumption of any food. In contrast, Lubransky et al. [43] reported a lower postprandial glycemic peak concentration for kiwi preload compared to rice preload, which may be due to the lower glycemic index of fruit and its fiber content [44]. However, the effects of higher fiber intake on glycemic control are still controversial among diabetic patients. It has been recommended that an individual consume food rich in soluble fiber before a meal rather than consume it as a snack [45]. Nevertheless, this discrepancy needs caution and a confirmed study in a wider population, including those with impaired glucose tolerance to reflect the beneficial effect of manipulating fruit intake on blood glucose control.

This within-subject study design may reduce the errors associated with individual difference since each subject served as their own baseline. However, since the order of the study was in sequence, the treatment order may affect the result. Nevertheless, possible confounding factors have been found to not affect the outcomes of this study. Furthermore, a small portion size of fruit was used. A higher portion of fruit can be used to optimize the significant effect of fruit intake on the measurement outcomes [46,47]. A further limitation was self-reported physical activity of the subjects. Previous study has found a significant effect of exercise on suppression of appetite [48]. We only included subjects with low and moderate physical activity. Even though there were no directly measured levels of physical activity, the protocol was briefly explained to the subjects and they were asked to maintain their regular physical activity across the study. 

## 5. Conclusions

The result of this study suggests that the sequence of fruit consumption has a significant effect on satiety as indicated by GLP-1 and further reduction of subsequent energy intake by 18.5%. Consumption of fruit before a meal was more likely to lead to satiation than after a meal or no fruit. Hence, consumption of fruit before a meal suppresses appetite and could potentially help in weight regulation. However, there was no significant difference in the sequence of fruit consumption on blood glucose concentration. More research is needed to confirm the potential benefits of the timing of fruit intake on satiety and blood glucose control in both the short and long term and beyond a healthy population.

## Figures and Tables

**Table 1:** Energy intake during test meal, after test meal, and total energy intake daily for three sessions.

Parameter	Mean ± SE	Intervention Effect		
		P	Partial eta, ηp^2^	Power
**Test meal, kcal**		0.419	0.049	0.156
Control	806 ± 46			
Before a meal	754 ± 46			
After a meal	787 ± 53			
**Test meal + apple, kcal**		0.504	0.037	0.128
Control	806 ± 46			
Before a meal	825 ± 46			
After a meal	857 ± 53			
**Subsequent energy intake, kcal**		0.031	0.195	0.660
Control	890 ± 55 ^a^			
Before a meal	725 ± 50 ^b^			
After a meal	786 ± 58			
**Total daily energy intake, kcal**		0.142	0.115	0.395
Control	2289 ± 59			
Before a meal	2156 ± 66			
After a meal	2255 ± 78			

Different letters within a column denotes significant difference (*p* < 0.05) with one-way repeated measure ANOVA, Bonferroni adjusted-pairwise comparison.

**Table 2:** Relationship between satiety level (iAUC Composite Satiety Score (CSS)) and appetite-related hormone.

Variable	iAOC Ghrelin		iAUC GLP-1		iAUC CCK8	
	r	p	r	p	r	p
**Incremental area under the curve CSS**						
Control	−0.573	0.016 *	−0.163	0.531	−0.244	0.346
Before a meal	−0.032	0.904	−0.297	0.247	0.173	0.506
After meal	−0.011	0.968	0.147	0.574	0.237	0.378

iAOC: incremental area over the curve; iAUC: incremental area under the curve. * Significant at *p* < 0.05 with Pearson correlation.
